# Axonal Energy Crisis and Calcium Phosphate Dysregulation as Pathogenesis of Optic Disc Drusen

**DOI:** 10.14336/AD.2024.0459

**Published:** 2025-09-30

**Authors:** Shweta Modgil, Tazbir Ahmed, Yaping Joyce Liao

**Affiliations:** ^1^Department of Ophthalmology, Stanford University, Palo Alto, CA 94303, USA.; ^2^Department of Neurology, Stanford University, Palo Alto, CA 94303, USA

**Keywords:** calcification, optic disc drusen, retinal ganglion cells, optic nerve, mitochondria, calcium, phosphate, ischemia, neuron

## Abstract

Soft tissue ectopic calcification is due to abnormal accumulation of calcium and phosphate outside the bone. It is the result of the failure of a complex, active, and highly regulated process - much of which is still not well understood. Some of our understanding of ectopic calcification come from studies on diseases such as atherosclerosis, aortic valve disease and kidney stone disease. In the eye, the most common causes of visual defects due to ectopic calcification include optic disc drusen (ODD) and age-related macular degeneration (AMD). In ODD, ectopic calcification occurs only in the most anterior, unmyelinated portion of optic nerve - the sole output of the eye and particularly susceptible to hypoxic and metabolic stress. In this article, we review the effects of hypoxia on mitochondrial function and calcium regulation and delineate the key processes likely involved in ectopic calcification. We propose a working hypothesis for ODD centering on the role of hypoxia-induced calcium and phosphate overload, mitochondrial dysfunction and osteogenic differentiation, leading to hydroxyapatite deposition and biomineralization.

## Introduction

Ectopic calcification or soft tissue biomineralization is an active and highly regulated process [1, 2]. While calcification is a normal process in bone, teeth, and the hypertrophic zone of growth plate cartilage, pathologic calcification occurs as a result of injury, inflammation, metabolic stress and aging, leading to common diseases such as vascular calcification in atherosclerosis, cardiac valve disease, and kidney stone disease [3-6]. Ectopic calcification of the eye is common, occurring (from front to back) as band keratopathy of the cornea, conjunctival concretions, calcification of the tendonous insertions of the eye, dacryoliths or stones in the lacrimal drainage system, optic disc drusen (ODD) of the optic nerve, and calcified macular drusen in age-related macular degeneration (AMD). The most devastating consequences of calcification of the eye occur in the retina and optic nerve because this process leads to irreversible vision defects. While retinal calcification in AMD affects the photoreceptor’s ability to capture visual information, optic nerve calcification in ODD impacts the transmission of information from the eye to the brain, where most of the visual processing and perception occurs in humans.

ODD is calcified yellowish extracellular deposits that only occur in the unmyelinated anterior optic nerve - a location that is easily visualized on clinical examination or ophthalmic imaging. These deposits are present in about 2.2% of general population [7]. Historically, ODD are often considered benign because, in the majority of cases, they are present without overt vision loss. However, these calcified deposits lead to severe crowding of the optic disc, the critical space where approximately 1.2 million retinal ganglion cell axons in the human eye converge and make a 90 degree turn to exit the globe - a location of high metabolic, vascular, and biomechanical stress [8]. This severe crowding leads to the so-called disc-at-risk in some people and increases risk for vascular events [9]. In fact, we now know that the majority of patients with ODD experience some visual field loss [10, 11], and ODD are the most important risk factors for young-onset non-arteritic anterior ischemic optic neuropathy (NAION) or optic nerve stroke, which typically occurs in the 5^th^ decade of life or beyond but, in patients with ODD, can occur as early as in the first 3 decades of life [12-15]. In fact, the most common cause of vision loss in ODD is NAION, which leads to sudden onset of visual field loss and, in about 15% of cases, can occur in the second eye, which is particularly devastating [10]. We do not yet know how to predict who is at high risk for vision loss, but ODD can remain relatively stable with minimal progression in many patients [16]. Other than optic nerve stroke, vision field defects can also progress slowly over years, as ODD evolving from deep, buried drusen in children to superficial, more visible drusen in teenagers and adults [17, 18]. Visible drusen are associated with more frequent visual field defects compared to buried drusen, even though there is no overt ischemic event [19].

ODD most commonly present as sporadic cases but can also rarely occur in a hereditary pattern in an autosomal dominant fashion. Family members of patients with ODD have a 10 times increased risk of having ODD [20]. ODD can also be associated with syndromic, neurodegenerative diseases of the eye and brain. For example, ODD are associated with retinitis pigmentosa (RP), a genetic disorders characterized by a progressive loss of photoreceptor cells, pigmentary changes, and blindness [21]. A recent study showed that ODD are found in 30.0% of patients with RP - a figure 15 times higher than previously observed - suggesting there may be a pathogenic link between these two conditions [22]. ODD are also commonly found in patients with pseudoxanthoma elasticum (PXE), a rare condition with severe and progressive calcification of the Bruch’s membrane of the retina and vasculopathy leading to vision loss [23]. ODD are found in 24.5% of patients with PXE, including in patients with known ATP-binding cassette subfamily C member 6 (ABCC6) mutations [24]. ODD are also associated with a syndromic eye condition associated with membrane frizzled-related protein mutation called Syndrome of Nanophthalmos-Renititis Pigmentosa-Foveoschisis-Optic Disk Drusen [25]. Finally, ODD are also associated with neurodevelopmental disorders such as Joubert syndrome and ciliopathies [26, 27].

Drusen in optic disc were first reported in 1858 but have received little attention. ODD are only found in the anterior unmyelinated portion - an area with unique structural and functional adaptations [20, 21]. The passive transmission of action potential along the unmyelinated axons at this location necessitates high concentration of mitochondria along axons to meet the high energy demands [28, 29]. Consistent with the hypothesis that mitochondrial dysfunction is a key aspect of disease pathogenesis, the most striking ultrastructural finding in ODD is electron-dense deposits in the mitochondria [30]. Elemental analysis of drusen extracted from a 59-year-old patient with severe disease reveals that ODD consist of Ca (Ca) and phosphate (P) [31]. These findings raise the possibility that mitochondrial calcification and dysfunction are among the earliest pathological events in ODD, involving release of Ca and P-rich matrix vesicles (MVs) from mitochondria into the extracellular matrix [32].

**Figure 1.**
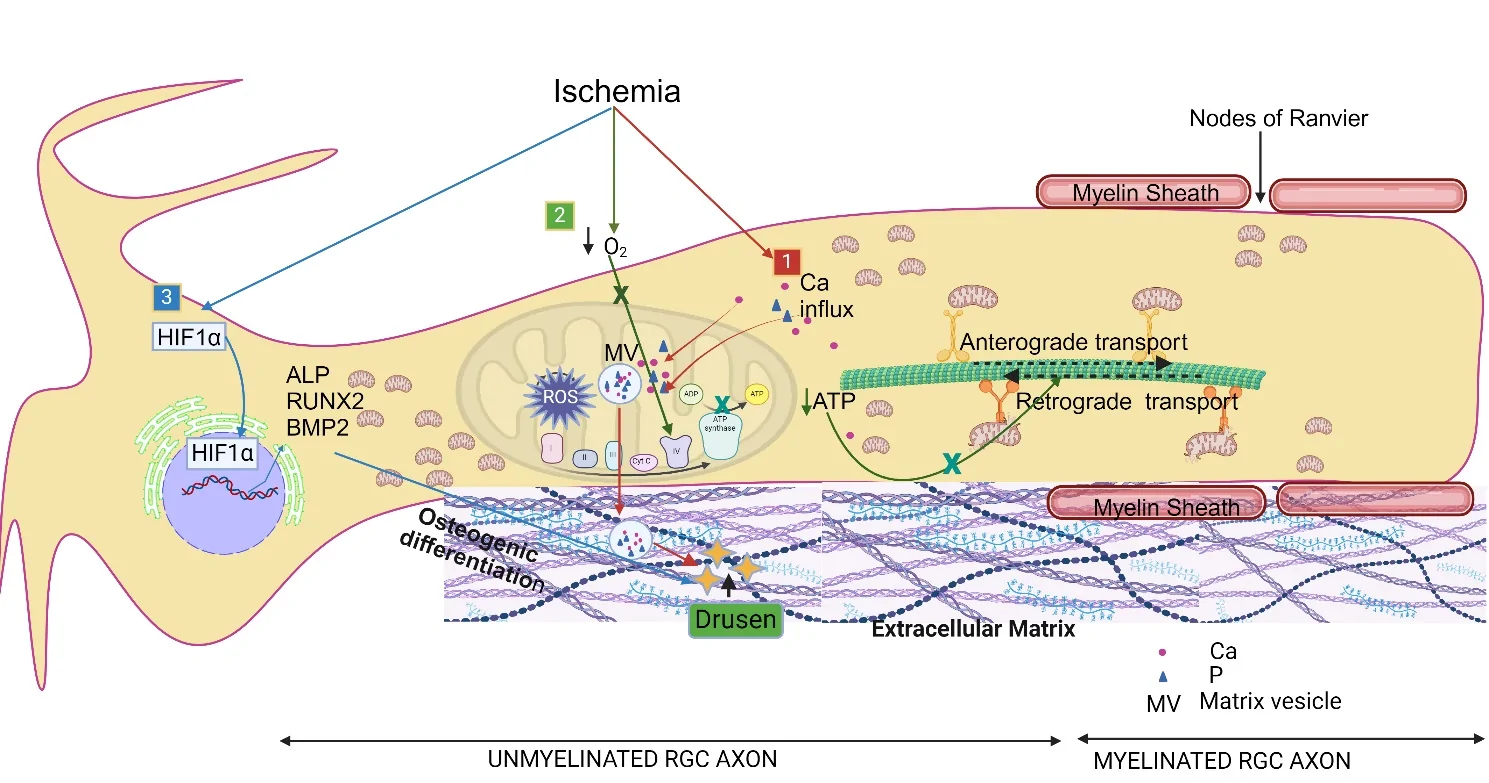
**Model of optic nerve calcification due to local axonal, energy crisis, and Ca and P dysregulation**. 1) Local hypoxic/ischemic insult leads to intracellular Ca overload and activation of various Ca-dependent channels and receptors. Intracellular influx of Ca activates calpains in the cytoplasm, reducing Ca-buffering capacity of the ER. Ischemia also promotes P uptake inside the cells by altering FGF23 expression. Ca and P overload in the cytoplasm opens mitochondrial Ca channels regulated by the MCU complex. Inside the mitochondria, Ca and P are compartmentalized inside the MVs to isolate oxidized protein, DNA and ROS and to prevent mitochondrial dysfunction. MVs interact with lysosomes and are released into the ECM by exocytosis. In the ECM, amorphous Ca-P deposits act as nidus for mineralization and drusen growth. 2) Lower O_2_ levels causing ATP deficit in neurons. Oxygen is terminal acceptor of oxidative phosphorylation (OXPHOS), and hypoxic/ischemic insult leads to low ATP production, impaired axonal transport, and clustering of abnormal mitochondria containing Ca-P complexes, further exacerbating the damage. ATP is a precursor for pyrophosphate (PPi), an important calcification inhibitor, and low ATP levels result in reduced level of PPi in the ECM, which can accelerate ectopic calcification. 3) Activation of HIF1α leads to upregulation of calcification genes promoting calcification. Abbreviations: ER: Endoplasmic reticulum; FGF23: Fibroblast growth factor 23; MCU: Mitochondrial calcium uniporter; MV: Matrix vesicle; ROS: Reactive oxygen species; ECM: extracellular matrix; ATP: Adenosine triphosphate; HIF1α: Hypoxia inducible factor 1α.

Ectopic calcification can occur in association with high or normal levels of systemic Ca and P. In conditions like uremia, there are high systemic levels of Ca and P, and a phenomenon called metastatic calcification can occur. In ODD patients, serum Ca and P levels are typically in the normal range. However, local increases in Ca and P levels at the optic nerve may contribute to a milieu prone to form Ca-P complexes. Because of the high metabolic stress at the anterior optic nerve, dystrophic calcification of the optic nerve [27] may occur due to local pathology. In this article, we will discuss the key events that can lead to biomineralization of the optic nerve, an important central nervous system white matter. We postulate that ischemia and extreme metabolic stress at this critical location leads to mitochondrial dysfunction, disrupted Ca homeostasis, release of MV and activation of osteogenic genes - conditions that prime formation of Ca-P complexes and hydroxyapatite deposition (Fig. 1).

## Regulation of Calcium and Phosphate

Although terms “mineralization” and “calcification” are used interchangeably in literature, it is crucial to note that Ca is necessary but not sufficient for inducing mineralization and that P is needed [58]. Saturated levels of Ca and P have a high propensity for precipitation and crystallization. For example, Ca-binding phospho-proteins can saturate the Ca-P concentration in the extracellular matrix, leading to ectopic calcification [33].

Most of the human body’s Ca and P are found in bone and teeth, and most Ca in the body is bound to proteins. Both Ca and P (P) come from dietary sources, and their levels in the body are highly regulated and strictly maintained within a physiological range through complex pathway and feedback control of sensors such as parathyroid hormone (PTH), calcitonin, vitamin D and fibroblast growth factor-23 (FGF-23). There are many excellent articles to further understand these regulatory pathways [34-39]. Calcification inhibitors such as pyrophosphate (PPi), osteopontin (OPN), fetuin-A, and matrix gamma-carboxyglutamic acid protein (MGP) actively chelate Ca, so it is relatively unavailable to bind with P, the first step in ectopic calcification [40, 41]. P is absorbed from the intestine by Na-dependent P transporters (NaP-IIb transporter and reabsorbed from the proximal tubule in kidney by NaP-IIa and IIc along with Pit-2; Type III Na-P cotransporters). Trafficking of Na-P transporters to and from renal tubular membrane is regulated by their interaction with sodium-hydrogen exchanger regulatory factors (NHERF1 and 3). High dietary P intake induces adaptation in this regulatory framework by stimulating PTH binding to PTHR initiating signaling cascade, which internalizes NaP-IIa and IIc receptors and reduces P absorption. High dietary P also leads to an increase in the level of FGF23, which activates fibroblast growth factor receptor (FGFR) and klotho, leading to increased urinary excretion of P [42]. Like PTH, FGF23 also diminishes NaP-IIa, NaP-IIc and Pit2 in the proximal tubule, causing urinary P excretion. FGF23 also reduces circulating level of 1,25(OH)_2_D3 (1alpha,25-dihydroxyvitamin D3, the active form of vitamin D) to reduce intestinal P absorption [39]. In summary, Ca and P are highly regulated to maintain normal calcification in bone and to prevent ectopic calcification in the soft tissues, and ectopic calcification is the result of pathologic breakdown of this complex process.

## Ischemia-induced calcium dysregulation and metabolic stress

We postulate that a likely pathologic process that leads to altered Ca homeostasis in the axons is hypoxic/ischemic insult.

Some earlier studies suggest a link between ischemia/reperfusion injury and calcification. Although the mechanism involving ischemia-induced pathological calcification are still not fully understood, studies by Kalantari et al. and Tzimas et al. point to the role of necrosis or apoptosis post ischemic insult [43, 44]. Neurons are especially vulnerable to ischemia because of glutamate receptor-mediated influx of extracellular Ca into the cell via opening of various Ca channels, activation of NMDA (N-methyl D-aspartate) receptors and prolonged membrane potential depolarization beyond critical threshold [45]. Ischemia also triggers the opening of other Ca channels such as transient receptor potential (TRP) channels and the TRPM2 and TRPM7 of TRPM superfamily, which are associated with ischemic Ca overload-related cell death [46]. Ischemia also results in metabolic stress and a metabolic shift from oxidative phosphorylation to glycolytic metabolism, leading to accumulation of lactate and hydrogen ion [47, 48], which further activates Ca-permeable, acid-sensing ion channels (ASICs) [49]. Because the anterior optic nerve is composed of long unmyelinated axons, which contain high concentration of mitochondria, local hypoxic/ ischemic insult can easily lead to energy crisis and metabolic deficits that can overwhelm and disrupt axonal homeostatic machineries far from the soma. Failure of calcification inhibitors (discussed below) at this critical location means the release of excessive Ca or cellular debris may serve as nidus for calcification.

Other than influx from extracellular space, Ca can also enter the cytoplasm from intracellular stores. The endoplasmic reticulum (ER) is a major intracellular reserve for Ca. The ER regulates the inflow-outflow of Ca via different receptors such as ryanodine receptors (RyR), sarcoplasmic/endoplasmic reticulum Ca ATPases (SERCA), and inositol triphosphate receptors (IP3Rs) [50]. Under normal conditions, Ca is taken up by the ER via SERCA-2 and released by RyR and IP3 receptors into the cytosol. Some Ca from ER is also released to the mitochondria for Ca-dependent signaling. The voltage-dependent anion channel (VDAC) on the outer mitochondrial membrane (OMM) allows for Ca entry into the mitochondria. From OMM, Ca moves to matrix via specific Ca channels via the mitochondrial Ca uniporter (MCU) complex present on the inner mitochondrial membrane (IMM). This dynamic exchange of Ca between the ER and mitochondria takes place at special contact sites called the mitochondrial associated membranes (MAM), where membranes of both organelles are in apposition and the IP3-VDAC complex scaffolding is highly favored [51]. This arrangement aids in the regulated flow of Ca ions between these organelles.

During hypoxic/ischemic insult and resultant mitochondrial stress, there is also a major metabolic shift from aerobic respiration to glycolysis, leading to a decrease in intracellular pH and production of lactic acid. To reduce acidosis, protons are pumped out of cell and sodium (Na) into the cell by the Na-H exchanger. This increase in intracellular Na leads to the reversal of the Na-Ca exchanger, which pumps Na out in exchange for Ca in, leading to increased Ca in the cytoplasm. Under conditions with elevated intracellular Ca, mitochondria behave as a secondary Ca reservoir and opening of the MCU shunts Ca into the mitochondria, restoring low Ca level in the cytoplasm [52]. Unfortunately, ischemia can also lead to downregulation of NCX and impaired ability to regulate intracellular Ca, leading to intracellular Ca overload, disrupted homeostatic mechanisms and irreversible damage to the neuron [53].

Regardless of the receptor types involved, the influx of Ca into the cytoplasm and subsequently into the mitochondria promotes expression of calcification genes. For example, persistent elevation of cytoplasmic Ca activates calpains, which are Ca-dependent proteases. Calpains then modify the phosphatase activity of calcineurin, which activates NFAT (nuclear factor of activated T cell), a regulatory transcription factor for osteogenic gene expression [54]. Furthermore, calpains cleave Ca regulatory proteins at the plasma membrane and ER, potentiating intracellular Ca levels. Ca dysregulation at ER by calpains also further increases mitochondrial Ca overload.

Ca plays a vital role in mitochondrial functioning; however, its effect is concentration dependent. Low Ca concentrations promote energy transduction, while elevated Ca levels lead to uncoupling of electron transport chain (ETC) [55]. Notably, Malyala et al. [56] found that it is not the free Ca levels in the matrix, but the Ca-P levels that affect the rate of ATP synthesis. They speculated that the Ca-P precipitates create a physical barrier isolating complex 1 from NADH and destabilizes it, causing energy distress. High Ca level alone does not favor MV formation as high Ca interferes with the potential gradient indispensable for survival, explained brilliantly in reviews by Shapiro et al. [57] and Carafoli et al. [58]. When P binds to free Ca, hydroxyapatite formation favors more Ca influx while maintaining the potential required. Thus, P levels are critical in formation of Ca-P complexes within the mitochondria.

The importance of P as a determinant for calcification is supported by studies in klotho/NaP-IIa double knock out mice. Studies in these animals indicat that high Ca and vitamin D alone are not sufficient to induce mineralization. Under normal P levels, calcification is prevented even at extremely high serum Ca and vitamin D levels [42]. The mitochondrial P carrier (PiC) protein encoded by SLC25A3 serves as a dominant P transporter into the mitochondria and is a part of the mitochondrial permeability transition pore [59]. P-rich diet in animals elevated levels of mitochondrial P carrier PiC, consistent with active transport to regulate and control high levels of P [60]. High P increases the abundance of PiC in mitochondrial fraction and genetic suppression of PiC prevented osteogenic differentiation and calcification of aortic rings in culture [61]. P as a key regulator is further supported by elevated FGF23, a master regulator of P levels, in renal injury patients [62]. Mice with renal ischemia/reperfusion (I/R) injury exhibit altered expression of many proteins involved in maintaining Ca, magnesium (Mg) and P levels, supporting the role of ischemia in calcification through mineral dysregulation [63]. It is noteworthy that renal ischemic injury is associated with both hyperphosphatemia and hypermagnesemia [64]. Interaction of these minerals are complex, earlier work has suggested high P promotes [65], while Mg inhibits mineralization [66]. More studies are needed to explore how these minerals influence the course of mineralization.

In summary, ischemia-induced Ca influx through opening of various ion channels leads to cytosolic Ca overload. Elevated Ca activates proteases, which affect Ca regulatory proteins, leading to Ca dysregulation. ER’s failure to buffer high Ca favors movement of Ca into the mitochondria. Ca entry plus movement of P through mitochondrial PiC lead to accumulation of these minerals inside the mitochondria promoting formation of Ca-P complex due to oversaturation.

## Impaired axonal homeostasis and ATP deficiency act as triggers for biomineralization

Neurons are selectively vulnerable to ATP deficit because the axons travel long distances to synapse with their targets. The maintenance of the axons relies on active axonal transport of neurotrophic factors, proteins, organelles, and synaptic vesicles and retrograde movement of waste material - processes that are all impacted by ATP deficit during ischemia. Mitochondria are the most important source of ATP, generated via oxidative phosphorylation (OXPHOS). with oxygen serving as the terminal electron acceptor in this chain. Complex IV, also known as cytochrome c oxidase (COX), oxidizes cytochrome c and transfers the electrons to oxygen [67]. Ischemia causes metabolic slowdown hampering ATP generation. Ischemia affects ATP production, disrupts retrograde transport [68], and inhibits mitophagy (the removal of dead and dysfunctional mitochondria), leading to accumulation of dysfunctional mitochondria in the axon [69]. In ischemia, anterograde transport of newly synthesized and healthy mitochondria to the axons is halted, further exacerbating axonal energy crisis. A study by Zheng et al. shows that ischemia leads to differential bidirectional mitochondrial transport in ischemic neurons [69]. Oxygen-glucose deprivation in neurons inhibits both anterograde and retrograde mitochondrial transport with particularly significant impact on anterograde transport. Reperfusion leads to recovery of retrograde but not antegrade movement implying that reperfused tissue never gets back to the normal stage as some damage is almost irreversible. Disruptions in bidirectional transport in neurons, as seen in retinal ischemia/reperfusion studies, may have severe consequences [70].

Pathological accumulation of dysfunctional proteins such as tau, amyloid beta, α-synuclein in the neurons are hallmark of neurodegenerative diseases [71]. Abnormal synthesis/degradation of these proteins is previously considered the most likely cause of these aggregates, but we now know that impaired axonal transport of proteins is key part of pathogenesis and so these diseases are being studied in the light of disrupted axonal transport. Advances in technology to perform large scale sequencing studies have highlighted the importance of axonal transport in neurodegenerative diseases. Impaired axonal transport following cerebral ischemia is postulated as a risk for Alzheimer’s disease [72]. Ischemia disrupts continuous flow of material to and from the soma by disassembling the axonal cytoskeleton [73]. It can therefore be postulated that axonal transport disturbances due to ATP deficit promote accumulation of dysfunctional mitochondria which act as nidus for calcification. At the same time, replenishment of new mitochondria at the site of ischemia is also diminished by slow anterograde transport feeding the loop of low ATP supply and its associated transport. Ischemic reperfusion injury affects mitochondrial dynamics with increased fission and inhibited fusion observed in renal I/R reported by simultaneous enhanced expression of dynamin-related protein and downregulation of mitofusin 2 (MFN2) gene. Mitochondrial biogenesis is also adversely affected during ischemia; peroxisome proliferator-activated receptor gamma coactivator-1 alpha (PGC-1α), the master regulator for biogenesis, is inhibited during ischemia [74]. This unbalanced mitochondrial fission, fusion, and biogenesis contribute to mitochondrial dysfunction, potentially predisposing mitochondria to membrane potential alterations and Ca accumulation, favoring calcification [74-76].

Beyond its role as energy currency, ATP also serves as a substrate for the ectonucleotide pyrophosphatase 1 (eNPP1) producing PPi, a key anti-mineralization molecule. Recently, the ABCC6 gene, associated with ectopic mineralization, is implicated in regulating ATP efflux. ABCC6 is believed to present on plasma membrane but contrary to this belief, Martin and coworkers reported it to be bound to mitochondrial membrane [77]. Although the localization of ABCC6 is currently disputed, its role in ATP efflux is evident from studies in zebrafish, where ABCC6 protein deficit causes ocular calcification [78]. The exact mechanism for this action is not yet known but the regulation of ATP release by ABCC6 explains how it may be involved in calcification. ABCC6 loss may reduce PPi in the extracellular matrix, lifting the negative regulation of mineralization. Indirect evidence of ATP/PPi conversion came from a study where M2 macrophage polarization in response to vascular calcification shows anti-calcification properties that are attributed to enhanced capacity to synthesize extracellular ATP, which is converted to PPi via eNPP1 [79]. Generation of PPi is thus indirectly dependent on ATP production further emphasizing the impact of ischemia on calcification through lowered anti-mineralization agents such as PPi. In summary, neurons, reliant on active axonal transport for various cellular components, suffer significantly from energy deficits during ischemia, resulting in the accumulation of dysfunctional mitochondria loaded with excess Ca and P. In addition, low PPi in the extracellular matrix due to low ATP efflux further tips anti-mineralization proteins off balance, favoring mineralization of already supersaturated levels of Ca and P.

## Ischemia activates the HIF-Osteogenic axis

Hypoxia inducible factor 1α (HIF) emerges as a pivotal regulator in response to hypoxia following ischemic insult. Alpha subunits of HIF are sensitive to oxygen and respond rapidly to low oxygen levels. Under normoxia, prolyl hydroxylase domain-containing enzymes mark HIF for proteasomal degradation by hydroxylating conserved proline residues [80]. Hydroxylated α subunits are subsequently ubiquitinated by VHL (von-Hippel-Lindau) and degraded [81]. Hypoxia impedes hydroxylation of HIF, enhancing its stability and translocation to nucleus. In the nucleus, HIF acts as a transcription regulator for numerous proteins. Diseases associated with hypoxemia and/or hypoxia, such as asthma, chronic obstructive pulmonary disease, and obstructive sleep apnea have been linked with increased vascular calcification [82, 83]. Similarly, diabetes patients are vulnerable to coronary artery calcification and their serum HIF1 levels are positively correlated to coronary artery calcification [84]. In diabetes, the formation of advanced glycation end products (AGEs) due to high glucose levels have been shown to promote the osteogenetic differentiation of vascular smooth muscle cells (VSMCs) [85]. A recently deciphered mechanism revealed that AGE-associated calcification involves the pyruvate dehydrogenase kinase 4 (PDK4)/HIF pathway. AGE stimulates HIF, which, in turn activates PDK4 and induces calcification [86]. Additionally, both chronic kidney disease and pulmonary arterial hypertension are associated with RUNX- induced vascular calcification which is related to HIF activation [87, 88].

HIF has been implicated in osteogenic differentiation and bone development [89]. Hypoxia leads to HIF activation and osteogenic trans-differentiation in vascular smooth muscle cells in presence of P [90]. Interestingly, high P alone can induce HIF even in normal level of oxygen, suggesting that P induces activation of HIF axis via a currently unknown mechanism independent of oxygen [90]. In contrast, Balogh et al. show that hypoxia is an independent calcification inducer in VSMCs [91]. Animal models of vascular calcification in the aorta under hypoxic conditions indicate that hypoxia alone is sufficient to induce HIF activation and soft tissue calcification. This was further corroborated by hypoxia-induced differentiation of mesenchymal cells to osteogenic lineage by activation of HIF-STAT3 pathway [92]. Direct evidence for HIF involvement in high P induced calcification is provided by Toth et al. [93]. Daprodustat, a prolyl hydroxylase inhibitor, accelerates calcification in high P-stimulated VSMCs by preventing degradation of HIF by VHL. These effects are reversed when HIF is downregulated, consistent with the direct action of HIF on the calcification processes. Inhibition of HIF may be a promising therapeutic approach. Capsaicin-induced degradation of HIF through SIRT 6 inhibits osteogenic differentiation under high P condition [94]. Similarly, estrogen driven vascular calcification inhibition is also reported to act through destabilization of HIF signaling [95]. Together, these studies link ischemia and its regulator HIF to osteogenic differentiation of soft tissue leading to calcification.

## Matrix vesicle release as early event for ectopic calcification

MVs are a type of extracellular vesicles (EVs) involved in the early stages of mineralization. Mitochondria and lysosomes are involved in MV biogenesis [96]. Electron microscopy studies have shed light on mitochondrial origin of these vesicles with electron dense granules containing Ca-P in these organelles [97]. Mitochondria containing these vesicles are also seen in autophagosomes [98, 99]. Mechanistically, elevated mitochondrial Ca induces formation of vesicles that are pinched off from mitochondria and then delivered to lysosomes. Although MVs have been shown to serve as nucleation foci for microcalcification as they contain hydroxyapatite (HAP) crystals [100], the mechanisms favoring HAP formation and evasion of protective barriers that normally inhibit ectopic calcification remain poorly understood. Wuthier et al. speculate that nucleation may be initiated in the inner leaflet of the MV membrane, based on the observations of amorphous crystals appearing first there [101]. Abundance of phosphatidylserine and phospha-tidylinositol in MVs and the high affinity of acid phospholipids for Ca suggest a potential role of these lipid entities in HAP formation. A recent in silico study reveals that phosphatidylserine complexes moderate these Ca-P interactions and stabilization, providing support for this hypothesis [102].

## Role of mitochondria-derived vesicles

Mitochondria-derived vesicles (MDVs) are important part of mitochondrial quality control and emerge early during mitochondrial stress. During oxidative stress, these vesicles serve as pre-mitophagy endogenous mechanisms to isolate oxidized cargo such as damaged proteins, DNA, and other contents and fragments of mitochondria to protect essential proteins from further oxidative damage. At physiological conditions, mitochondria are highly efficient in maintaining the balance between ROS (reactive oxygen species) generation and scavenging by antioxidants [103]. When this balance is tipped off, such as during ischemia, excessive ROS is generated which stimulates the formation of MDVs to mitigate the effect of damaged proteins, DNA, and other contents and to prevent complete shutdown of mitochondria [104]. Instead of degrading whole mitochondrion, upregulation of MDVs offers a mechanism to isolate and remove dysfunctional content to restore homeostasis [105]. Interestingly, the generation of MDVs is not exclusively a stress response, and MDVs have been observed in both physiologic and pathological conditions and the controlled release of MDVs can help promote mitochondrial dynamics and cellular well-being. In pathological conditions, a large number of MDVs can be generated in a way that is harmful to cellular homeostasis [106]. In this scenario, instead of being degraded by lysosomes, MDVs are trafficked to cell surface by linking with multivesicular bodies. The content inside MDVs, which may contain high concentrations of Ca-P complexes, is then secreted as exosomes into the extracellular space, where it may initiate signaling cascades or elicit immune responses that can prime extracellular hydroxyapatite formation [107].

Proteomics studies indicate that 10% of exosomes may be of mitochondrial origin, which reinforces the contribution of this secretory pathway for MDV release [108, 109]. Extracellular vesicles containing mitochondrial proteins and enzymes are elevated in the serum from patients with degenerative diseases like Parkinson’s disease and sarcopenia, consistent with MDVs as a major source of these EVs [110]. Bone, cartilage, and dentin routinely secrete MV containing the required enzymes, lipids along with Ca-P complexes for mineralization [111, 112]. The MV contains amorphous Ca-P, which can become crystalline hydroxyapatite when released and bound to ECM.

As discussed earlier, ischemia results in Ca and P overload in the mitochondria, so it is plausible that high Ca stimulate biogenesis of MDVs and is trafficked to the lysosomes, similar to the process observed in bone mineralization [111]. Lysosomes, acting as degradation machinery fuse with plasma membrane, lead to exocytosis of content to extracellular matrix [113]. Once released into ECM, vesicular content interacts with ECM proteins, inducing mineralization. The specific mechanisms by which hypoxia augments the calcification process beyond increasing the number of MDVs remain largely unknown. One possibility is that hypoxia prevents the lysosomal degradation of proteins essential for mineralization within MDVs. This protection can be exhibited through glycosylation of these proteins, rendering them immune to lysozymes. Lysosomal membrane proteins use a similar mechanism to evade lysosomal degradation and are highly glycosylated [114]. Tissue-nonspecific alkaline phosphatase (TNAP) and annexin in the MDVs are glycosylated, supporting this hypothesis [115]. Although direct evidence linking hypoxia to the stability and functionality of proteins in MDVs is lacking, a recent study links hypoxia and upregulation of HIF-1α to the elevation of GLT8D1 (glycosyltransferase 8 domain containing 1) levels in cancer cells. GLT8D1 promotes N-linked glycosylation of CD133 (Prominin1, a tumor initiating protein) rendering it resistant to lysozymes negatively affecting its degradation [116]. Taken together, ischemia triggers various down stream effects which either can independently or in combination led to mineralization. The complex cascade of reactions following ischemia and crosstalk between various molecular mechanisms makes it difficult to develop target therapeutics.

## Working hypothesis for axonal ischemia-induced ODD formation

**Figure 2.**
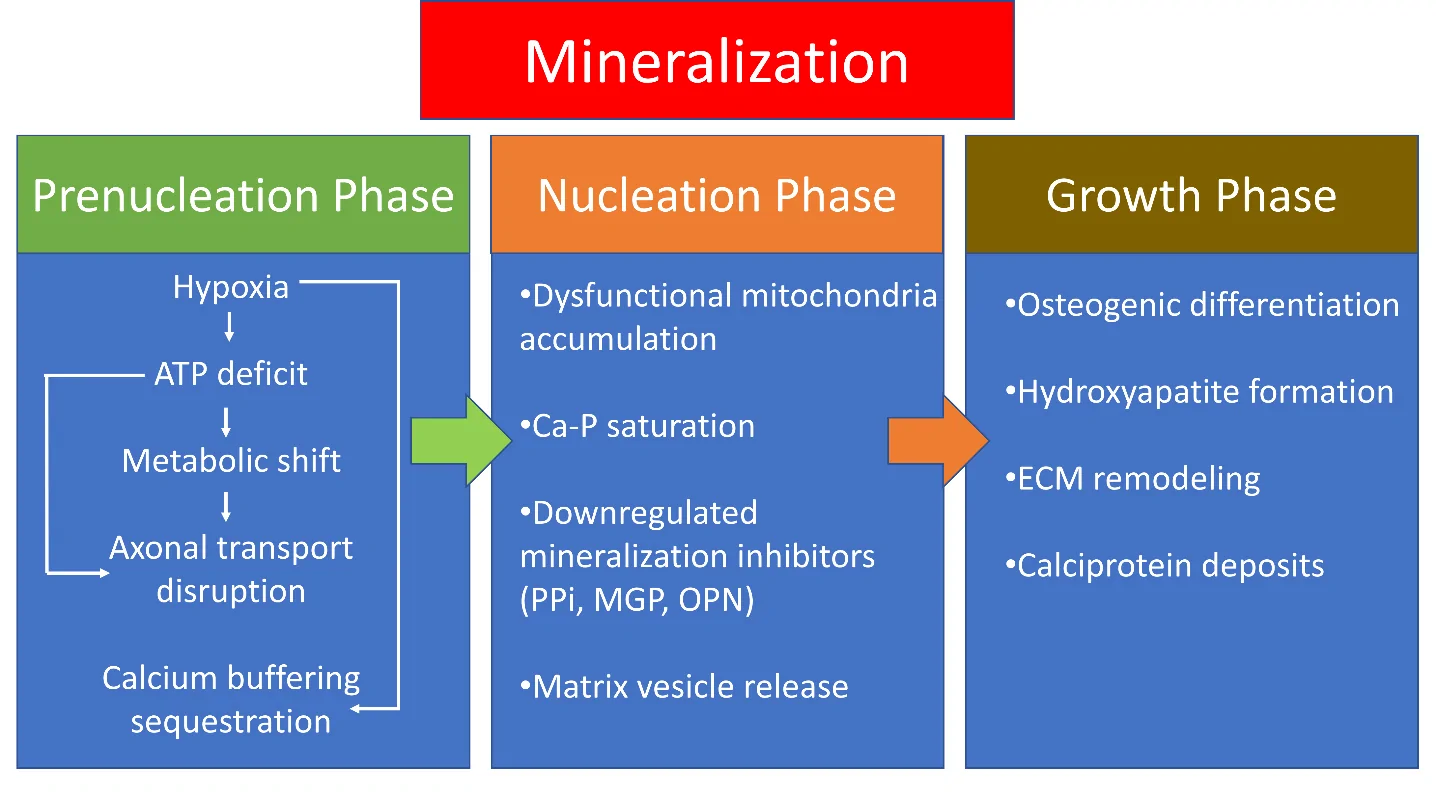
**Proposed mechanism for optic nerve calcification**. Biomineralization is a complex process that can be divided into three stages. *The prenucleation phase* creates the microenvironment that leads to saturation levels of Ca and P. This may be triggered by local axonal energy crisis, disrupting bi-directional axonal transport and interfering with Ca and P regulatory machineries. At this stage, there is no visible ODD; however, the molecular changes are already set for pathologic calcification. *Nucleation phase* involves ATP deficiency and Ca overload, leading to accumulation of Ca-P complexes. Mitochondrial function and dynamics are altered, further exacerbating energy crisis, and ATP deficit lowers the level of important calcification inhibitor PPi, favoring calcification. This energy crisis triggers the release of MVs containing saturated Ca-P and other proteins into the extracellular matrix (ECM). *Growth phase*, Ca-P in combination with ECM proteins serve as nidus for formation of crystalline hydroxyapatite (HAP), which enlarges over time to form visible ODD. This process may involve coalescence of many vesicles, some of which contain abnormal mitochondria containing Ca-P. Abreviations: PPi: Pyrophosphate; MGP: Matrix gamma carboxyglutamic acid protein; OPN: osteopontin.

We propose a working model for ODD formation, which divides the mineralization process into three phases: prenucleation (no visible mineralization), nucleation (where Ca-P microdeposits may or may not appear), and growth (visible calciprotein particles in the extracellular space) (Fig. 2). In the prenucleation phase, transient ischemia in this metabolically demanding region can trigger events that, if unchecked, lead to pathological mineralization. We posit that ischemia is a primary factor driving local Ca and P imbalance, the initial molecular events driving drusen formation. While we do not know when the prenucleation events occur, we do know that this can occur in the first decade of life or possibly even *in-utero* [117]. Local hypoxic/ischemic insult or activation of the HIF-osteogenic pathway early in life may prime the optic nerve to eventually form ODD over years.

In the nucleation stage, mitochondrial dysfunction plays a crucial role. Given that prelaminar optic nerve is unmyelinated and has numerous mitochondria to support the high energy demand; disruption of mitochondrial dynamics may selectively impact this region. Mitochondria have their own regulatory mechanism (fusion and fission) to maintain functionality [118]. When these mechanisms fail, unhealthy mitochondria are targeted to mitophagy and removal. For clearance, damaged mitochondria must be transported back to the soma for mitophagy, which is an ATP-dependent process. Energy crisis during hypoxic/ischemic insult means damaged mitochondria are not transported and disposed, accumulating in the prelaminar zone, creating a chokepoint for drusen formation. Another reason for selective vulnerability of the unmyelinated optic nerve could be the absence of oligodendrocytes and protective myelin sheath, making it easier for mitochondria to extrude out of the axons, serving as a nidus for extracellular mineral deposition. Recent studies using murine model of autoimmune diseases affecting the central white matter show that nanorupture in the axolemma is an important mechanism for Ca entry into the axon [119, 120], and unmyelinated axons may be selectively vulnerable to rupture and dysregulation of Ca and P in the axon.

Failure of mitochondrial function and dysregulation of intracellular Ca and P lead to formation and secretion of MVs laden with Ca-P complexes, which can act as nidus for drusen formation. It is important to recognize the complexity of this process and undersatand that mitochondria are not the sole organelles involved. ER and the endo/lysosomal compartments are also important in this process. Additionally, just as Bruch’s membrane is important in ectopic calcification of the retina in AMD, extracellular matrix at the optic nerve head likely also plays a significant role and need to be considered to understand why ODD form at this location.
